# MaxEnt-Based Predictions of Suitable Potential Distribution of *Leymus secalinus* Under Current and Future Climate Change

**DOI:** 10.3390/plants14020293

**Published:** 2025-01-20

**Authors:** Shimeng Zhao, Zongxian Zhang, Changyu Gao, Yiding Dong, Zeyao Jing, Lixia Du, Xiangyang Hou

**Affiliations:** Key Laboratory of Efficient Forage Production Mode, Ministry of Agriculture and Rural Affair, College of Grassland Science, Shanxi Agricultural University, Jinzhong 030801, China; zsmsxau@163.com (S.Z.); zongxian.zhang1@gmail.com (Z.Z.); 18434761726@163.com (C.G.); nice_1998@126.com (Y.D.); jing_zeyao@163.com (Z.J.); dulixia0328@126.com (L.D.)

**Keywords:** *Leymus secalinus*, species distribution modeling (SDM), shared socioeconomic pathway (SSP) scenarios, environmental variables, suitable area

## Abstract

Grassland degradation is a serious ecological issue in the farming–pastoral ecotone of northern China. Utilizing native grasses for the restoration of degraded grasslands is an effective technological approach. *Leymus secalinus* is a superior indigenous grass species for grassland ecological restoration in northern China. Therefore, the excavation of potential distribution areas of *L. secalinus* and important ecological factors affecting its distribution is crucial for grassland conservation and restoration of degraded grasslands. Based on 357 data points collected on the natural distribution of *L. secalinus*, this study employs the jackknife method and Pearson correlation analysis to screen out 23 variables affecting its spatial distribution. The MaxEnt model was used herein to predict the current suitable distribution area of *L. secalinus* and the suitable distribution of *L. secalinus* under different SSP scenarios (SSP1-26, SSP2-45, and SSP5-85) for future climate. The results showed the following: (1) Mean diurnal temperature range, annual mean temperature, precipitation of the wettest quarter, and elevation are the major factors impacting the distribution of *L. secalinus*. (2) Under the current climatic conditions, *L. secalinus* is mainly distributed in the farming–pastoral ecotone of northern China; in addition, certain suitable areas also exist in parts of Xinjiang, Tibet, Sichuan, Heilongjiang, and Jilin. (3) Under future climate change scenarios, the suitable areas for *L. secalinus* are generally the same as at present, with slight changes in area under different scenarios, with the largest expansion of 97,222 km^2^ of suitable area in 2021–2040 under the SSP1-26 scenario and the largest shrinkage of potential suitable area in 2061–2080 under the SSP2-45 scenario, with 87,983 km^2^. Notably, the northern boundary of the middle- and high-suitability areas is reduced, while the northeastern boundary and some areas of Heilongjiang and Jilin are expanded. The results of this study revealed the suitable climatic conditions and potential distribution range of *L. secalinus*, which can provide a reference for the conservation, introduction, and cultivation of *L. secalinus* in new ecological zones, avoiding the blind introduction of inappropriate habitats, and is also crucial for sustaining the economic benefits associated with *L. secalinus* ecological services.

## 1. Introduction

Grasslands, which cover 40% of the land area, are an essential global biodiversity repository, providing enormous material and non-material benefits to humans [1,2,3]. However, grassland degradation is very serious globally and is increasing in many areas, with about 49% of the world’s grasslands being degraded to varying degrees to date, undermining their ability to maintain biodiversity, ecosystem services, and benefits to people [4]. China is one of the countries with the richest grassland resources in the world. Grassland resources not only provide an important material basis for the development of livestock husbandry, but also play an important ecological function in maintaining soil and water, preventing winds and stabilizing sands, nourishing water, protecting biodiversity, and maintaining the balance of ecosystems [5]. The farming–pastoral ecotone of northern China extends from the northeast to the southwest, following the line of the well-known Great Wall of China. It is a typical farming–pastoral zone in history, and nowadays, and it is also an important ecological security barrier in the middle-eastern region of China and an essential water conservation belt in the Beijing–Tianjin–Hebei region, which is of vital significance for the ecological security of the country [6]. Over the past half-century, ecological and environmental problems such as grassland degradation, land sanding, and salinization have arisen due to climate change and anthropogenic phenomena such as over-cultivation and overloaded grazing [7,8]. This has made the farming–pastoral ecotone one of the most serious ecological problems in China. In China, there are currently 264 million hectares of grassland, with 70% of the area being degraded [9,10]. Since the late 1990s, the Chinese Government has carried out a variety of ecological protection projects and policies to alleviate the degradation of grassland ecosystems in the farming–pastoral ecotone. These include the ‘Returning Ploughland to Forests and Grassland Project’, the ‘Beijing-Tianjin Wind and Sand Source Control Project’, the ‘Three-North Protective Forests Protection Project’, and the ‘Grassland Ecological Protection Subsidy and Incentive Policies’ [11,12,13]. The implementation of these measures has partially changed the way grasslands are utilized and curbed the deterioration of the ecological environment in the farming–pastoral ecotone. This has mitigated the trend of grassland degradation and improved the ecological environment. However, despite efforts to reverse the trend of grassland degradation, grassland protection and restoration remain challenging tasks [14,15].

The demand for high-quality grass species is rising due to grassland protection and construction projects, leading to stricter selection criteria [16]. While exotic breeds are often introduced, they struggle to adapt to local climates, increasing restoration costs and invasion risks [17]. In contrast, native plants, being well-adapted to local conditions, are resilient, cost-effective, and easier to maintain, making them the preferred choice for ecological construction [18]. Particularly in fragile farming–pastoral ecotones, selecting suitable native grass species can significantly enhance ecological functions and support grassland restoration [19].

*Leymus secalinus*, a perennial herb of the *Poaceae* family, is mainly distributed in the western part of Northeast China, Hebei, Shanxi, Ningxia, Sichuan, Qinghai, Xinjiang, and other provinces. It is an excellent pasture grass, with drought resistance, saline–alkaline tolerance, trampling resistance, and strong adaptability, making it ideal for ecological restoration in farming–pastoral ecotones [20]. Its underground rhizomes are effective for wind-breaking, sand-fixing, and soil and water conservation [21,22], playing an important role in combating desertification, improving saline–alkaline land, and restoring the ecological environment [23,24]. Additionally, *L. secalinus* holds significant economic and ecological value [25] and is a valuable genetic resource for wheat improvement due to its stress resistance and genetic diversity [26,27]. Therefore, it is crucial to study its distribution and exploitation for the protection and restoration of grassland in the farming–pastoral ecotone.

In recent years, climate change, ecological deterioration, human interference, and the “low spiking, low fruiting, low germination” issues of *L. secalinus* have severely limited its practical application. Collecting and conserving *L. secalinus* germplasm resources are a vital material basis for livestock production, breeding, and ecological restoration. Systematic surveys and scientific sampling across its distribution area are needed to protect and utilize these resources effectively. Studies like FRPS and others [28,29,30] have detailed its distribution, while classifying potential areas based on environmental factors can guide conservation and planting zoning. Although research has explored the genomic, chemical, and biological control of *L. secalinus* [31,32,33,34,35,36,37], its ecological suitability, spatial distribution, and response to climate change in China have not been previously reported. Addressing these gaps will enhance understanding of its distribution and adaptability under future climate change scenarios.

Species distribution models (SDMs) [38] are a method for estimating the distribution of species in geospatial space based on the actual distribution of species and are a mathematical model based on species distribution data and environmental variables, which have been broadly applied to the prediction of species’ potential areas [39,40] and can still give good results when species distribution data are not complete [41,42]. With the advance of mathematical modeling methods and geographic information systems (GIS), numerous species distribution models have been established, and the popular species distribution models include the Maximum Entropy (MaxEnt) model, Bioclimatic Model (BIOCLM), Genetic Algorithm for Rule-set Production (GARP), and Generalized Linear Model (GLM) [43]. Among them, the MaxEnt model is the most widespread [44,45,46,47]. Compared to other species distribution models, the MaxEnt model exhibits excellent forecasting ability and accuracy. It is particularly effective in situations where species distribution data are scarce (e.g., when distribution points are few or sample sizes are small), as it can quantitatively describe the potential distribution area of species through screening of major ecological factors, so as to realize the simulation of species distribution. Therefore, it has a wider application in predicting the potential distribution of invasive species and assessing the impact of climate change on species and has become a mainstream prediction model [48,49,50,51,52].

In this work, we simulated the potential distribution of *L. secalinus* in China by means of the MaxEnt model. Specifically, this work aims to (1) predict the potential geographic distribution of *L. secalinus* in China under current climatic conditions, (2) identify the key environmental factors affecting the distribution of *L. secalinus* and quantitatively describe the environmental conditions suitable for its survival, and (3) predict the natural trends and changes in the suitable habitats of *L. secalinus* in future climate scenarios and provide recommendations for *L. secalinus* management and use.

## 2. Materials and Methods

### 2.1. Study Area

*L. secalinus* is a perennial herbaceous plant of the family *Poaceae*, primarily distributed in Xinjiang, Gansu, Qinghai, Shaanxi, Sichuan, Nei Mongol, Hebei, Shanxi, and the northeast in China, with a wide range of habitats and strong adaptability. The geographical coordinates of its main natural distribution areas are 75° E~128.9° E and 29.4° N~49.08° N.

Our study area includes the farming–pastoral ecotone, which is traditionally the main agricultural area to the east and south, and the main grassland animal husbandry to the west and north. In the vicinity of the farming–pastoral ecotone, however, farming and grazing co-exist and intermingle.

### 2.2. Collection of Occurrence Data

**Figure 1:**
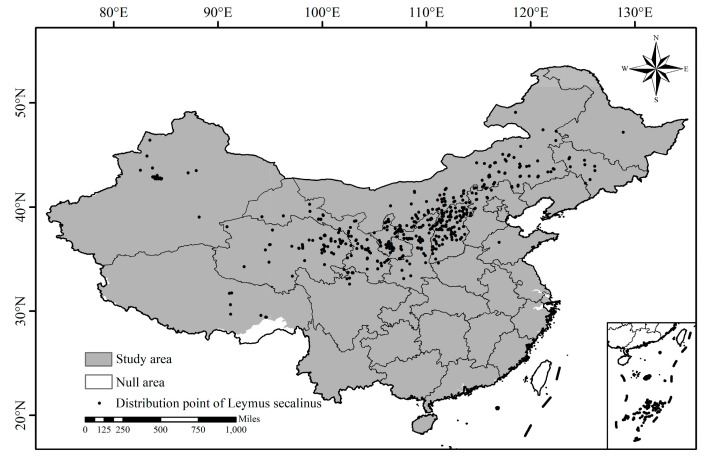
Distribution of *Leymus secalinus* in China.

Data on the geographic distribution of *L. secalinus* are mainly from field investigation data in 2022–2023 and relevant distribution information recorded in the literature, the latter included records from the Chinese Virtual Herbarium (CVH), and a total of 493 distribution points of *L. secalinus* were collected. To avoid model overfitting, distribution points were filtered using a threshold of 100 m. When the distance between multiple points was less than 100 m, one point was randomly retained while the others were removed. Finally, 357 distribution points of *L. secalinus* in China were obtained (Figure 1).

Note: Taiwan Province and some other regions are not included in this study (the same as below).

### 2.3. Environmental Variables

This study used 52 environmental variables, including 19 bioclimatic factors, 30 soil factors, and 3 topographic factors (Table S1). Climate data were sourced from the World Climate Database (https://worldclim.org (accessed on 13 January 2024)). The environmental variables data include current (1970–2000) and future climate data (2021–2040, 2041–2060, 2061–2080, 2081–2100), with the future climate condition data containing data for three shared socioeconomic pathways (SSP1-26, SSP2-45, and SSP5-85) [53]. The 19 commonly used bioclimatic factors are denoted Bio1-Bio19. Soil factors were obtained from FAO SOILS PORTAL (https://www.fao.org/soils-portal/en/ (accessed on 13 January 2024)), and in this study, all the data above and below ground of the soil were separated to form a file in tiff format to ensure the spatial continuity of the data. The topographic factors include three factors: DEM (elevation), Slope, and Aspect. The Digital Elevation Model (DEM) data were obtained from the geospatial data cloud platform (https://www.gscloud.cn/ (accessed on 17 January 2024)), and Slope and Aspect data were obtained using the spatial analysis function of ArcGIS. The spatial resolution of all used environmental variables was transformed to 1 km^2^.

Due to the varying degrees of correlation between environmental variables, it is essential to analyze and address multicollinearity to minimize its impact on predictive outcomes and the contributions of variables, thereby ensuring the model’s precision and accuracy. We considered the importance of the variables obtained by the jackknife method, quantitatively evaluated the effect of environmental factors on the geographic distribution of *L. secalinus*, and tested the correlation of environmental factors using Pearson (Figure S1) [54], and environmental variables with high correlation (r ≥ 0.8) and low contribution rates or 0 were removed [55,56,57]. Ultimately, 23 environmental factors were retained (Table 1).

### 2.4. Geographic Data Sources

The map data for this study are 1:1 million Chinese maps and administrative division maps downloaded from the National Basic Geographic Information Database (http://www.ngcc.cn/dlxxzy (accessed on 17 January 2024)) as the base map for analysis.

### 2.5. MaxEnt Model

The MaxEnt model is a sophisticated machine learning algorithm that emulates the probability of a species’ existence from only the existing data and environmental variables, based on the principle of maximum entropy. This study employed MaxEnt version 3.4.1. Of the distribution point data, 75% was randomly selected as training data for model construction, while the other 25% was used as testing data for model validation. To reduce model uncertainty, the Bootstrapping method was used to perform 10 replicate runs for each subsample type, and the results were averaged, with the number of iterations set to 500 and background points set to 10,000. The jackknife test was employed to obtain the contribution percentage of environmental variables. Jackknife experiments were performed on all environmental variables to determine the main environmental factors influencing the distribution of *L. secalinus*. The MaxEnt model uses the ROC curve of the subject’s work characteristics to measure model prediction accuracy, and the area enclosed by the ROC curve and the abscissa is known as the AUC value (ranging from 0.5 to 1.0), which is recognized as the optimal detection index of model accuracy [45,58]. The higher the AUC value, the better the simulation results. Based on the AUC value, model performance can be categorized as follows: 0.9–1, extremely accurate; 0.8–0.9, accurate; 0.7–0.8, moderately accurate; 0.6–0.7, general; and 0.5–0.6, failure [59].

### 2.6. Prediction of Suitable Area of L. secalinus

To more intuitively show the suitable habitat area and distribution characteristics, the model-predicted suitable habitat for *L. secalinus* was classified into four levels using Jenks natural breaks. The levels are as follows: In the high-suitability area (0.51–1), the distribution of *L. secalinus* is dominated by dominant species, and its habitat is especially suitable for *L. secalinus*. The middle-suitability area (0.26–0.51) has a large number of *L. secalinus* distribution, but *L. secalinus* is not a dominant species in the community. The low-suitability area (0.08–0.26) has a distribution of *L. secalinus*. The non-suitable area (0–0.08) is unsuitable for the growth of *L. secalinus*, and there may be no *L. secalinus* distribution.

## 3. Results and Analysis

### 3.1. Accuracy Evaluation of MaxEnt Model

**Figure 2:**
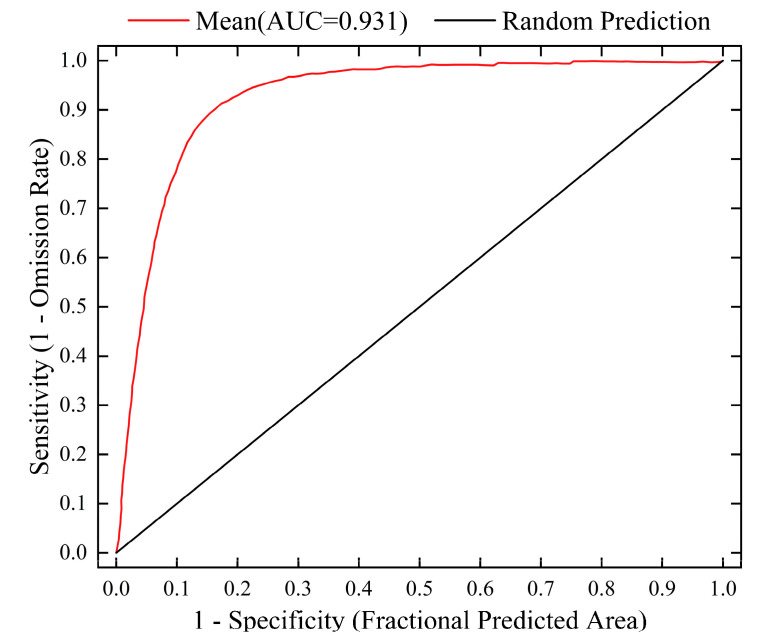
ROC curve of MaxEnt models for *L. secalinus*.

**Figure 3:**
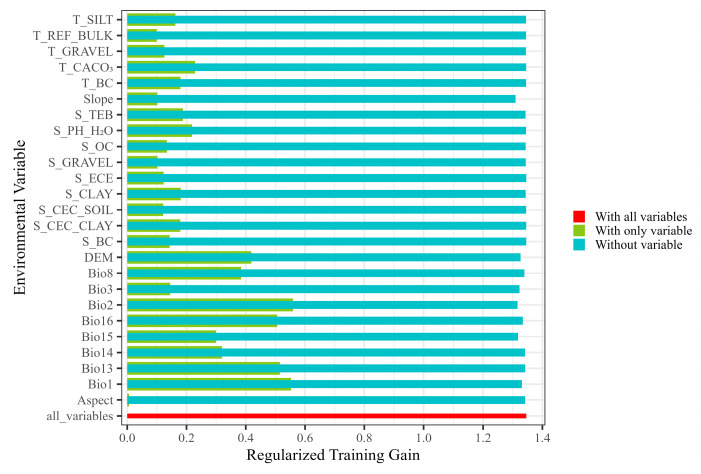
Regularized training gain of the MaxEnt model based on the jackknife test.

The accuracy of the MaxEnt model’s predictions for the potential suitable area of *L. secalinus* was evaluated using the ROC curve., and the ROC curve of the calculation results was obtained (Figure 2). It can be seen from Figure 3 that in this study, the average AUC of the data from the 10 trials’ data was 0.931 with a standard deviation of 0.003. This shows that the prediction results of the model have high accuracy and reliability and can reasonably simulate the distribution of *L. secalinus* in China, which provides conditions for further research.

### 3.2. Analysis of Important Environmental Variables

Based on the contribution rate and permutation importance of environmental variables in the MaxEnt model (Table 1), the contribution rates of Bio2, Bio1, Bio16, DEM, and Bio15 for modeling were 27.1%, 22.1%, 18.8%, 7.3%, and 7.2%, respectively, and the cumulative contribution rate was 82.5%. This indicated that Bio2, Bio1, Bio16, DEM, and Bio15 were the major environmental factors influencing the potential suitable area of *L. secalinus*. Among the permutation importance of environmental variables, the permutation importance values of Bio16, Bio2, DEM, Bio3, and Bio1 were 22.6%, 16.8%, 12.6%, 9.4%, and 9.2%, respectively, with a cumulative importance of 70.6%, indicating that these five environmental variables play a key role in the modeling process. The results of the jackknife test (Figure 3) show that when only this factor is present, the five environmental factor variables that have the largest effect on the regularization training gain are Bio2, Bio1, Bio13, Bio16, and DEM. This shows that these environmental variables contain more useful information compared to others. The gain value of Aspect normalization training is the lowest, indicating that it has the least influence on the distribution of *L. secalinus*.

Looking at it from a holistic point of view, under the current climatic conditions, Bio2 (mean diurnal temperature range), Bio1 (annual mean temperature), Bio16 (precipitation of wettest quarter), and DEM (elevation) are the main environmental factors affecting the distribution of *L. secalinus*. The temperature factor had the greatest effect, followed by the precipitation factor, while topographic and soil factors have a relatively minor influence on the distribution of *L. secalinus*.

**Figure 4:**
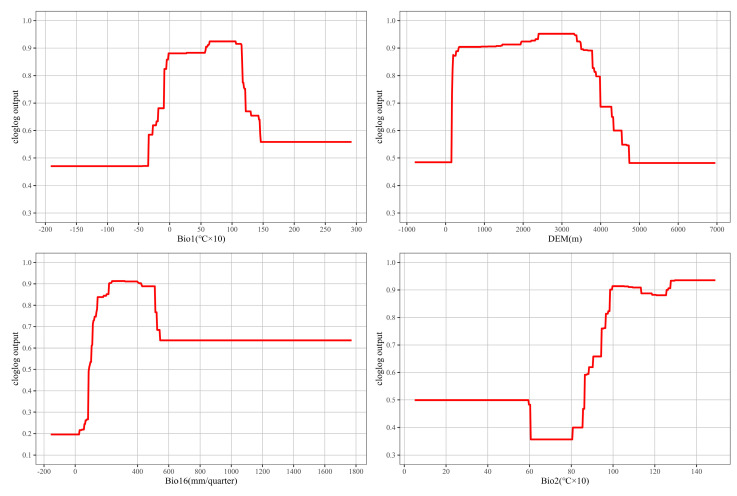
Response curve of main environmental variables in *L. secalinus* (Bio1, Bio2 units are °C × 10).

The species response curve reveals the relationship between environmental variables and the probability of species existence, and it shows biological tolerance to target species and habitat preferences [60]. It is generally believed that the occurrence probability threshold greater than 0.5 is regarded as the corresponding environment conducive to the growth of *L. secalinus* [61]. Based on response curves of four major climate variables (Figure 4), Bio1 of −3.3–11.6 °C, DEM of 160–3300 m, Bio16 of 90–400 mm, and Bio2 of 8.6–15 °C were suitable for the distribution of *L. secalinus*.

### 3.3. The Suitable Distribution Area of L. secalinus in China Under the Current Climate Scenario

**Figure 5:**
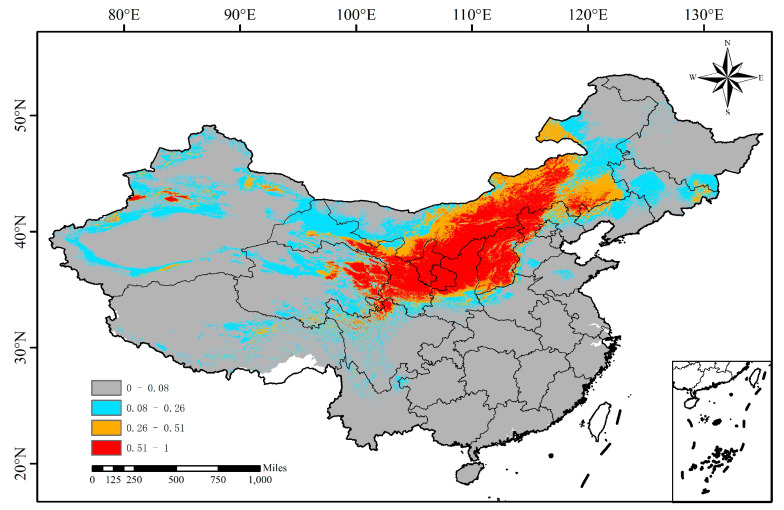
Predictions of the potentially suitable area of *L. secalinus* under current climate conditions based on the MaxEnt model.

Using the MaxEnt model for simulation, classification of “presence and absence” according to MTTS thresholds, and using ArcGIS to map a visual analysis chart. Under the current climatic conditions, the distribution of suitable areas of *L. secalinus* in China is shown in Figure 5. The red areas represent the high-suitability area of *L. secalinus*, the orange areas represent the medium-suitability area, the blue areas represent the low-suitability area, and the gray areas represent the non-suitable area. As shown in Figure 5, the potential suitable distribution areas of *L. secalinus* are located in 18 provinces and cities in Northwest China and North China, mainly concentrated in the range of the farming–pastoral ecotone.

The highly suitable areas of *L. secalinus* are primarily distributed in Shanxi Province, Inner Mongolia, Shaanxi, Ningxia, Gansu, eastern Qinghai, western and northeastern Xinjiang, northwestern Sichuan, northwestern Hebei, western Liaoning, and sporadically in central Tibet, western Henan, northwestern Beijing, and eastern Jilin, accounting for 8.89% of China’s total area (Table 2).

The distribution range of the suitable area of *L. secalinus* is usually located around the high-suitability area, accounting for 7.08% of China’s total area.

The low-suitability areas of *L. secalinus* are primarily located in parts of north-central and southern Xinjiang, southeastern Tibet, central and southern Qinghai, northwest and southern Gansu, western and eastern Inner Mongolia, the middle of Shaanxi, northwestern Henan, southwestern and Northeastern Hebei, the middle of Shandong, northern Liaoning, central and eastern Jilin, and western and southern Heilongjiang, with small portions located in northern Yunnan and western Guizhou, accounting for 15.75% of China’s total area.

The other provinces and cities are non-suitable for planting *L. secalinus*, accounting for 68.28% of China’s total area.

### 3.4. The Suitable Distribution Area of L. secalinus in China Under Future Climate Scenarios

**Figure 6:**
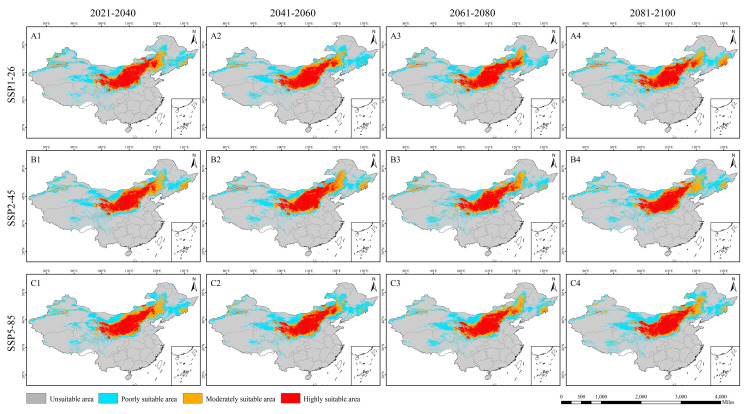
Potential distribution of *L. secalinus* under the SSP1-26 (**A1**–**A4**), SSP2-45 (**B1**–**B4**) and SSP5-85 (**C1**–**C4**).

**Figure 7:**
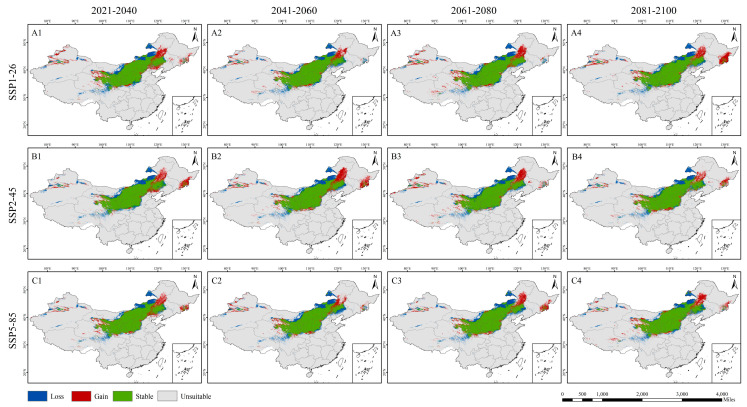
Potential changes in high- and medium-suitability areas for *L. secalinus* considering different SSPs. Changes in distribution highlighted in different colors; for gain (range expansion), red; loss (range contraction), blue; unsuitable, grey; and stable, green. (**A1**–**A4**) SSP1-26, (**B1**–**B4**) SSP2-45 and (**C1**–**C4**) SSP5-85.

This study is based on the three socioeconomic pathways proposed by the IPCC (SSP1-26, SSP2-45, SSP5-85); the MaxEnt model was used to predict the possible geographical distribution of *L. secalinus* in 2021–2040, 2041–2060, 2061–2080, and 2081–2100 (Figure 6). The results of this study found that the range of potential suitable areas of *L. secalinus* under current climatic conditions and different climatic scenarios in the future is basically the same as the range of the farming–pastoral ecotone. In addition, there are also suitable areas in Xinjiang, Tibet, Sichuan, Heilongjiang, and parts of Jilin. The overall behavior of the potentially suitable areas for *L. secalinus* in the three simulated scenarios was largely similar, with slight differences in the details of the predictions (Table 2). We observed that among all scenarios and years, SSP1-26 had the highest rate of expansion of suitable area in 2021–2040 and SSP5-85 in 2081–2100, with 4.29% and 3.47%, respectively (Figure 6(A1,C4)), and in all years of SSP2-45, the suitable areas were reduced compared to the current period, with 2061–2080 having the greatest shrinkage of potential suitable areas at 4.19% (Figure 6(B3)). In predicting potential changes in high- and medium-suitability areas, we also observed a significant decrease in potential suitability areas along the northern limit and an increase in eastern Inner Mongolia (including Xing’anmeng and southern Hulunbeier), southern Heilongjiang (including Mudanjiang and the western part of Jixi City), and eastern Jilin (Figure 7). A trend towards the northeast was present.

## 4. Discussion

### 4.1. Discussion on MaxEnt Model to Predict the Current Distribution of L. secalinus

Despite differences in simulation results from different climate models, the MaxEnt model remains an essential research tool for evaluating and forecasting past and future changes in species’ geographic distributions [62]. Based on the MaxEnt model combined with ArcGIS, this study predicted the potential distribution area of *L. secalinus*, providing an intuitive understanding of the current spatial distribution structure and the distribution of suitable areas in China. This research lays a basis for future studies on the geographic distribution of *L. secalinus*, as well as provides scientific evidence for future management and exploitation of *L. secalinus*. This study analyzed the percentage contribution of each ecological factor to the model and employed Pearson correlation coefficient analysis to address the collinear effect.

This study used the ROC curve to evaluate the species distribution model [63,64], running the MaxEnt model ten times with all data and taking the average AUC value from these runs to assess the model’s accuracy [65,66]. It is generally believed that a higher AUC value suggests a closer alignment between the model’s predicted species distribution and the actual distribution. The findings show that the average AUC value of the model is 0.931 with a standard deviation of 0.003, which proves that the prediction results are accurate and the model fitting is excellent, which can reflect the potential geographic distribution of *L. secalinus* in China extremely accurately.

The prediction results show that *L. secalinus* is primarily distributed in Shanxi, Inner Mongolia, Shaanxi, Ningxia, Gansu, Qinghai, Xinjiang, Sichuan, Hebei, and Northeast China, which is in line with the actual situation of the major production areas of *L. secalinus* in China. According to the relevant literature and the investigation report of *L. secalinus* resources in recent years [28,29], the comparison between the prediction results and the distribution status of *L. secalinus* in China shows that the existing distribution conforms to the prediction results and is mostly in the high-suitability area, which confirms the reliability of the MaxEnt model predictions.

The MaxEnt model, with its high predictive accuracy, flexibility, and reliance solely on species presence data, has been widely applied in the fields of ecological conservation and resource management. Additionally, MaxEnt can quantify the importance of environmental variables, assisting managers in identifying key driving factors and providing a scientific basis for optimizing management strategies. However, the application of MaxEnt also has certain limitations, such as potential uncertainties in predictions caused by data biases, uneven sampling distributions, or the omission of critical variables. Moreover, the selection of environmental variables in model construction requires comprehensive consideration of multiple factors, including climatic, ecological, biological, and anthropogenic influences. Therefore, as an efficient predictive tool, the outputs of MaxEnt should be integrated with actual ecological and social factors to enhance the scientific rigor and feasibility of management decisions.

### 4.2. Discussion on the Main Ecological Factors

Climate is a determining factor in the distribution of species, with changes in species distribution area being the most direct and clear reflection of climate change [67]. Climate, historical distribution, topography, soil, and other factors have significant effects on species distribution at different spatial scales. Among these factors, temperature and precipitation emerge as the predominant drivers affecting the growth of most plants [68]. Temperature factors affect species distribution by affecting plant photosynthesis and respiration, while precipitation conditions also constrain plant growth and development. Among the 23 environmental variables utilized for modeling in this study, the 4 key factors restricting the current potential suitable distribution of *L. secalinus* are mean diurnal temperature range (8.6–15 °C), annual mean temperature (−3.3–11.6 °C), precipitation of wettest quarter (90–400 mm), and elevation (160–3300 m), accounting for 75.3% of the total contribution rate. These factors reflect the temperature conditions, precipitation conditions, and topographic conditions required for the growth of *L. secalinus*. This is consistent with the habits of *L. secalinus*: *L. secalinus* is not only slightly fond of humidity, but also tolerant of cold and drought, and has a wide range of adaptation [69,70]. The increased frequency of extreme weather events in the future is expected to result in significant temperature fluctuations [71], whereas *L. secalinus* is suitable for living in habitats with cooler temperatures and is more widely distributed on the Qinghai–Tibet Plateau [72]. The climatic conditions of alpine grassland make *L. secalinus* seriously stressed by alpine hypoxia, intense solar radiation, and rapid climate change. The ability of *L. secalinus* to adapt to the alpine growth environment indicates that it has its own cold tolerance mechanisms. Large temperature fluctuations may lead to a reduction in or even loss of suitable areas, so proper precautions need to be taken.

In addition, precipitation is also a crucial variable affecting the establishment and survival of species, with higher precipitation generally being favorable for seedling growth; however, the connection between moisture availability and species distribution is contingent upon local conditions and the ecological demand of related species. Notably, excessive water in the soil reduces the root respiration rate, thus limiting plant growth. The response curves showed that the suitable area for *L. secalinus* increases up to a precipitation threshold of 400 mm in the wettest quarter. However, an increase in precipitation beyond this threshold results in a sharp decline in the suitable area. For instance, the main climatic factors affecting the distribution pattern of the recently reported *L. chinensis*, which is in the same genus as *L. chinensis* [73], are the precipitation of the coldest quarter, precipitation of the driest quarter, and precipitation of the driest month. The distribution value increases as cold-season precipitation rises. When precipitation during the cold season reaches 44.53 mm, the distribution value decreases with the increase in precipitation; precipitation during the driest quarter and driest month show the same pattern with their distribution values, which is the same as the results of the present study and both indicate the impact of precipitation on the establishment and survival of species and that the expansion or reduction of natural populations depends on future precipitation.

### 4.3. Discussion on the Suitable Area of Species Under SSP Model

A growing body of research has found that under the global climate change scenario, some species (*Acorus calamus* [74], *Pistacia chinensis* [75], *Phoebe sheareri* [76], *Leersia hexandra* [66]) will likely expand their distribution area, and for most species, their distribution area will decrease [47,60,77,78]. This study shows that *L. secalinus* suitable areas performed relatively consistently in different climate change scenarios, with generally similar distributional ranges. Projections of potential suitable areas showed a loss in all years under the SSP2-45 scenario, but only up to a maximum of 4.19% from the current (2061–2080), with the smallest range loss in 2081–2100; in contrast, the SSP1-26 (2021–2040) and SSP5-85 (2081–2100) scenarios had the largest range expansions (4.29% and 3.47%, respectively). Therefore, these projections suggest minimal overall changes in distribution patterns, indicating that *L. secalinus* has strong stress resistance to future climate change, has certain adaptability, and can survive and maintain its distribution to a greater extent. In addition, most of the high- and medium-suitability areas will not change considerably in the future and will be identical to the current period, with the exception of a partial shrinkage of the northern boundary. Previous studies have shown that under future climate scenarios, the extent of some species’ suitable areas will not change considerably, and the overall pattern will be consistent with the current distribution [79,80]. These findings are consistent with the conclusions of this study that the ranges of some suitable areas will remain unchanged with climate change, indicating that these species are expected to retain the largest potential distribution area in the current and future climate change scenarios.

This study explains changes in the geographic distribution of suitable *L. secalinus* habitat by considering the expansion, contraction, and preservation of suitable areas. Under future climate change scenarios, the general trend of changes in the distribution of suitable *L. secalinus* is consistent. The areas that have been stably distributed include Shanxi, Shaanxi, Ningxia, Gansu, Inner Mongolia, Qinghai, and some areas of Hebei, indicating that these areas are conducive to growth and maintain the distribution of *L. secalinus*. The expanding areas are located in parts of Jilin and Heilongjiang in the northeast, as well as in parts of eastern Inner Mongolia. The shrinking area is located at the northern boundary of the current suitable area, and the geographic distribution changes are more significant, suggesting that *L. secalinus* is not adapted to this climatic environment and is sensitive to climate change, which may be attributed to the increase in precipitation and the rise in temperature in North China and Northwest China [81]. Measures should be taken to protect the existing *L. secalinus* resources in these areas to prevent the loss of valuable germplasm resources.

In conclusion, this study offers valuable information on the potential distribution of *L. secalinus* and provides a basis for conservation and management initiatives. It successfully predicted the potential distribution area of *L. secalinus* and revealed the main geographical distribution area of *L. secalinus* in northern China. A comprehensive analysis of various environmental factors identified the main climatic variables that affect species distributions. By considering the impact of different climate change scenarios on species distributions, it will be possible to develop more favorable and adaptive management strategies.

The shortcomings of this study are as follows: (i) the distribution modeling did not take into account non-environmental factors, such as light, wind direction, wind speed, human disturbance, biological interaction, etc.; (ii) *L. secalinus* is distributed in Russia, North Korea, and Japan, in addition to China, and the distribution points of *L. secalinus* obtained in this study were limited; and (iii) the projections under different shared socioeconomic pathways (SSPs) may not fully capture the complexity of future climate scenarios, and this study primarily focuses on short-term climate projections (up to 2100). Regrettably, we could not collect complete data on these factors, which should be taken into account in future studies to more fully consider the effects of non-environmental factors, socioeconomic changes, and longer time scales, integrating them into the distribution model, with more long-term and continuous scientific investigation and statistics, to further enhance the accuracy of the model and reduce the variability from the actual distribution area, to make better-informed decisions on managing and utilizing L. secalinus.

## 5. Conclusions

In this study, the MaxEnt model was applied to successfully predict the suitable distribution area of *L. secalinus* based on different environmental factors. Our results show that under the current climate scenario, *L. secalinus* is mainly distributed in the farming–pastoral ecotone of northern China, and the adaptation range is quite wide. The distribution of *L. secalinus* is mainly affected by the mean diurnal temperature range (Bio2), annual mean temperature (Bio1), precipitation of the wettest quarter (Bio16), and elevation (dem). Although the distribution area of *L. secalinus* will change slightly under different scenarios in the future, the overall distribution range is not expected to change significantly, and *L. secalinus* will migrate to the northeast in the future. The predicted results of this study can provide considerable reference value for the exploration and utilization of *L. secalinus* resources, help to understand the distribution law of *L. secalinus*, promote the protection and resource utilization of *L. secalinus*, promote the management work, and improve the ecological and economic value of *L. secalinus*.

## Figures and Tables

**Table 1:** Contribution and permutation importance of environmental variables affecting the distribution of *L. secalinus*.

Variable	Percent Contribution/%	Permutation Importance/%
Bio2	27.1	16.8
Bio1	22.1	9.2
Bio16	18.8	22.6
DEM	7.3	12.6
Bio15	7.2	6.2
Bio8	3.5	3.9
Bio13	3.1	2.3
Slope	2.9	9
Bio3	2.9	9.4
Bio14	2.3	4.2
S_OC	0.4	0.4
S_CLAY	0.4	0.7
S_PH_H_2_O	0.3	0.1
Aspect	0.3	0.8
S_TEB	0.3	0.4
S_GRAVEL	0.2	0.2
T_REF_BULK	0.2	0.3
T_GRAVEL	0.2	0.2
T_BS	0.1	0.2
T_SILT	0.1	0.2
S_CEC_CLAY	0.1	0
T_CACO_3_	0	0.1
S_CEC_SOIL	0	0.2

**Table 2:** Potential suitable area of *L. secalinus* in China under future scenarios.

Scenario	Period	Non-Suitable Area	Lowly Suitable Area	Moderately Suitable Area	Highly Suitable Area	Suitable Area Change Ratio
	current	68.28	15.75	7.08	8.89	
SSP1-26	2021–2040	66.92	17.05	7.00	9.03	+4.29%
	2041–2060	68.58	16.57	6.36	8.49	−0.95%
	2061–2080	68.56	15.62	6.80	9.02	−0.88%
	2081–2100	68.22	15.76	7.60	8.42	+0.19%
SSP2-45	2021–2040	69.20	15.46	7.27	8.07	−2.90%
	2041–2060	68.70	15.31	6.87	9.12	−1.32%
	2061–2080	69.61	15.17	6.96	8.26	−4.19%
	2081–2100	68.34	16.49	7.09	8.08	−0.19%
SSP5-85	2021–2040	67.74	16.66	7.20	8.40	+1.70%
	2041–2060	68.47	17.04	5.89	8.60	−0.60%
	2061–2080	69.24	15.49	6.99	8.28	−3.03%
	2081–2100	67.18	17.37	6.65	8.80	+3.47%

## Data Availability

The original contributions presented in this study are included in the article/Supplementary Materials; further inquiries can be directed to the corresponding authors.

## Supplementary Materials

The following supporting information can be downloaded at https://www.mdpi.com/article/10.3390/plants14020293/s1: Table S1. A list of environmental variables. Figure S1. Spatial autocorrelation heat maps for (a) 19 contemporary climate data, (b) 19 future climate data, (c) 30 soil data.
