# Supplementary material for: MaxEnt-Based Predictions of Suitable Potential Distribution of Leymus secalinus Under Current and Future Climate Change
Source: Plants (Basel). 2025 Jan 20;14(2):293. doi: 10.3390/plants14020293 (PMC11768666; doi:10.3390/plants14020293)
Supplement: Supplementary file 1 [file plants-14-00293-s001.zip › plants-3442458-supplementary.pdf]

Table S1. A list of environmental variables

Classification	Index	Description	Units
	Bio1	Annual Mean Temperature	℃×10
	Bio2	Mean Diurnal Range	℃×10
	Bio3	Isothermally	%
	Bio4	Temperature Seasonality	SD×100
	Bio5	Max Temperature of Warmest Month	℃×10
	Bio6	Min Temperature of Coldest Month	℃×10
	Bio7	Temperature Annual Range	℃×10
	Bio8	Mean Temperature of Wettest Quarter	℃×10
	Bio9	Mean Temperature of Driest Quarter	℃×10
Bioclimatic variables	Bio10	Mean Temperature of Warmest Quarter	℃×10
	Bio11	Mean Temperature of Coldest Quarter	℃×10
	Bio12	Annual Precipitation	mm
	Bio13	Precipitation of Wettest Month	mm
	Bio14	Precipitation of Driest Month	mm
	Bio15	Precipitation Seasonality	Coefficient of Variation
	Bio16	Precipitation of Wettest Quarter	mm
	Bio17	Precipitation of Driest Quarter	mm
	Bio18	Precipitation of Warmest Quarter	mm
	Bio19	Precipitation of Coldest Quarter	mm
	T_GRAVEL	Topsoil Gravel Content	% weight
	T_SAND	Topsoil Sand Fraction	% weight
	T_SILT	Topsoil Silt Fraction	% weight
	T_CLAY	Topsoil Clay Fraction	% weight
	T_REF_BULK	Topsoil Reference Bulk Density	Kg/dm^3
	T_OC	Topsoil Organic Carbon	% weight
	T_PH_H_2O	Topsoil pH (H_2O)	-log (H^+)
	T_CEC_CLAY	Topsoil CEC (clay)	cmol/kg
	T_CEC_SOIL	Topsoil CEC (soil)	cmol/kg
	T_BS	Topsoil Base Saturation	%
	T_TEB	Topsoil TEB	cmol/kg
Soil data	T_$CACO_3$	Topsoil Calcium Carbonate	% weight
	T_$CASO_4$	Topsoil Gypsum	% weight
	T_ESP	Topsoil Sodicity (ESP)	%
	T_ECE	Topsoil Salinity (Elco)	dS/m
	S_GRAVEL	Subsoil Gravel Content	% weight
	S_SAND	Subsoil Sand Fraction	% weight
	S_SILT	Subsoil Silt Fraction	% weight
	S_CLAY	Subsoil Clay Fraction	% weight
	S_REF_BULK	Subsoil Reference Bulk Density	Kg/dm^3
	S_OC	Subsoil Organic Carbon	% weight
	S_PH_H2O	Subsoil pH (H_2O)	-log (H^+)

	S_CEC_CLAY	Subsoil CEC (clay)	cmol/kg
	S_CEC_SOIL	Subsoil CEC (soil)	cmol/kg
	S_BS	Subsoil Base Saturation	%
	S_TEB	Subsoil TEB	cmol/kg
	S_CACO3	Subsoil Calcium Carbonate	% weight
	S_CASO4	Subsoil Gypsum	% weight
	S_ESP	Subsoil Sodicity (ESP)	%
	S_ECE	Subsoil Salinity (Elco)	dS/m
	DEM	Elevation	m
Terrain factors	slope	Slope	°
	aspect	Aspect	°

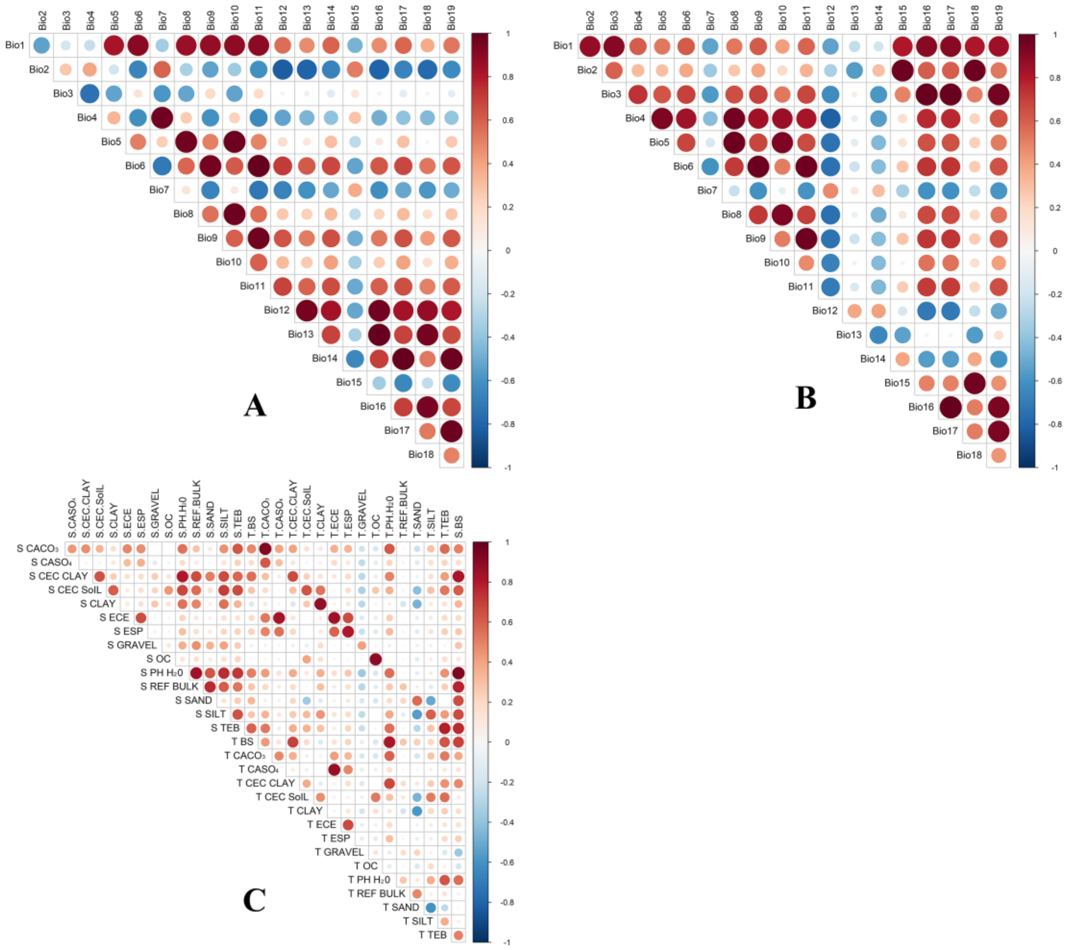

Figure S1. Spatial autocorrelation heat maps (a) 19 contemporary climate data, (b) 19 future climate data, (c) 30 soil data
